# From Zn to Mn: The Study of Novel Manganese-binding Groups in the Search for New Drugs against Tuberculosis

**DOI:** 10.1111/j.1747-0285.2010.01060.x

**Published:** 2011-02

**Authors:** Sarah L Williams, César Augusto F de Oliveira, H Vazquez, J Andrew McCammon

**Affiliations:** 1 Department of Chemistry & Biochemistry, University of California San DiegoLa Jolla, CA 92093-0365, USA; 2 Howard Hughes Medical Institute, University of California San DiegoLa Jolla, CA 92093-0365, USA; 3 Center for Theoretical Biological Physics, University of California San DiegoLa Jolla, CA 92093, USA; 4 Department of Pharmacology, University of California San DiegoLa Jolla, CA 92093-0365, USA

**Keywords:** drug design, drug discovery, molecular modeling, structure-based

## Abstract

In most eubacteria, apicomplexans, and most plants, including the causal agents for diseases such as malaria, leprosy, and tuberculosis, the methylerythritol phosphate pathway is the route for the biosynthesis of the C_5_ precursors to the essential isoprenoid class of compounds. Owing to their absence in humans, the enzymes of the methylerythritol phosphate pathway have become attractive targets for drug discovery. This work investigates a new class of inhibitors against the second enzyme of the pathway, 1-deoxy-d-xylulose 5-phosphate reductoisomerase. Inhibition of this enzyme may involve the chelation of a crucial active site Mn ion, and the metal-chelating moieties studied here have previously been shown to be successful in application to the zinc-dependent metalloproteinases. Quantum mechanics and docking calculations presented in this work suggest the transferability of these metal-chelating compounds to Mn-containing 1-deoxy-d-xylulose 5-phosphate reductoisomerase enzyme, as a promising starting point to the development of potent inhibitors.

Tuberculosis (TB) is a serious infectious disease caused by the *Mycobacterium tuberculosis* bacterium. It is a major cause of illness and death, and owing to a rise in HIV cases, the neglect of TB control programs, and an increase in drug resistance, the disease has resurged in recent years in well-developed countries and has exacerbated the TB problem in the lesser developed countries (1). Therefore, there is an urgent need for the development of new drugs and suitable therapeutic targets.

In most eubacteria, apicomplexans, and most plants, including the causal agents for diseases such as malaria, leprosy, and tuberculosis, the methylerythritol phosphate pathway (MEP, also known as the DOXP or non-mevalonate pathway) is the route for the biosynthesis of isopentenyl diphosphate and its isomer, dimethylallyl diphosphate (DMAPP), the common C_5_ precursors to isoprenoids (2–5). Isoprenoids comprise a large and diverse family of compounds with numerous vital and diverse functions, with involvement in processes such as respiration, electron transport, hormone-based signaling, and membrane stability (6,7).

The MEP pathway comprises nine enzymes (8,9), all of which have been identified as viable drug targets by genetic approaches (10,11) and are of particular interest owing to their absence in humans, who use the alternative mevalonate pathway (10,12). The 1-deoxy-d-xylulose 5-phosphate reductoisomerase (DXR) enzyme is the most studied of the pathway’s enzymes to date. This enzyme is involved in the second stage of the pathway, mediating the reversible intramolecular rearrangement and NADPH-dependent reduction of 1-deoxy-d-xylulose 5-phosphate (DXP) to 2-C-methyl-d-erythritol 4-phosphate (MEP) in the presence of a divalent metal ion (for which Mn^2+^ has shown to be the most effective (13)).

Drugs, such as fosmidomycin and its analogs, whose structure is similar to the natural substrate have been developed and shown to be efficacious against the *Escherichia coli* (14) and *Plasmodium falciparum* (15,16) DXR enzymes. As with the natural substrate, the inhibitors chelate the divalent metal ion present in the active site of the enzyme. However, as observed with the majority of antibiotics and chemotherapeutic agents, these inhibitors are ineffective *in vivo* against the *M. tuber* strain of the enzyme (17). In the case of fosmidomycin, the lack of potency has been attributed to the complex and hydrophobic nature of the mycobacterial cell wall and the absence of a cAMP-dependent glycerol-3-phosphate transporter preventing the uptake of such a small and highly charged molecule (18). Even in the absence of these resistance issues, such as in the treatment against the *P. falciparum* pathogen, the late recrudescence observed in clinical trials precludes the drug as a monotherapy, with efficacious treatment requiring it to be administered with clindamycin (15,16). Clinical studies have also shown that repeated and comparably high doses of the drug are required to achieve acceptable cure rates (15,16). Furthermore, although the hydroxamate moiety of fosmidomycin exhibits attractive metal-chelating properties, these compounds are associated with low availability, poor *in vivo* stability, and undesirable side-effects, making them often undesirable in the manufacture of drugs (19).

In this study, we propose an alternative metal-chelating group to hydoxamate, as a starting point for the development of a new class of inhibitors against the DXR enzyme. Cohen *et al.* have identified and synthesized a group of compounds, which are indicated to be successful alternatives to hydroxamate in the chelation of Zn^2+^, in the Zn-dependent matrix metalloproteinases (MMP) (20). The structures of the ligands featured in this study comprise hydroxypyridinones, hydroxpyridinethiones, pyrones, and thiopyrones (Figure 1). The study by Cohen *et al.* found these ligands to share similarities to the hydroxamate moiety in terms of their bidentate-chelate formation properties, with improved hydrolytic stability and biologic tolerance, and proposed an increase in affinity toward Zn.

**Figure 1:**
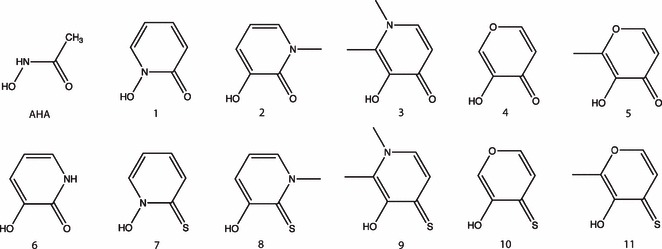
Structures of the metal-chelating groups (acetohydroxamic acid and metal-binding groups 1–11) examined in this study.

In this work, the computational techniques of quantum mechanics (QM) and QM-polarized docking calculations were used to study the potential of these metal-binding groups (MBGs) as potential Mn-binding moieties, as part of a search for a new class of inhibitors against the 1-deoxy-d-xylulose 5-phosphate reductoisomerase (*Mt*DXR) enzyme. This study provides promising results, indicating these compounds to possess similar or improved binding affinities against the *Mt*DXR enzyme, when compared to the reference hydroxamate-based compound, acetohydroxamic acid (AHA), therefore suggesting them to be good candidates for further development in an effort to produce potent inhibitors.

## Computational details

### Quantum mechanics calculations

Modeling ligand binding can be problematic using conventional methods, with issues such as polarization and charge transfer being inadequately accounted for in the usual forcefields. This can be a hurdle in the design of novel and potent inhibitors and is exacerbated with the presence of metal ions in the active site. In an attempt to determine whether the 12 proposed chelating moieties (Figure 1), taken from the study by Cohen *et al.*, would form chelates with a Mn^2+^ ion, QM calculations were used to predict the binding affinity.

**Figure 2:**
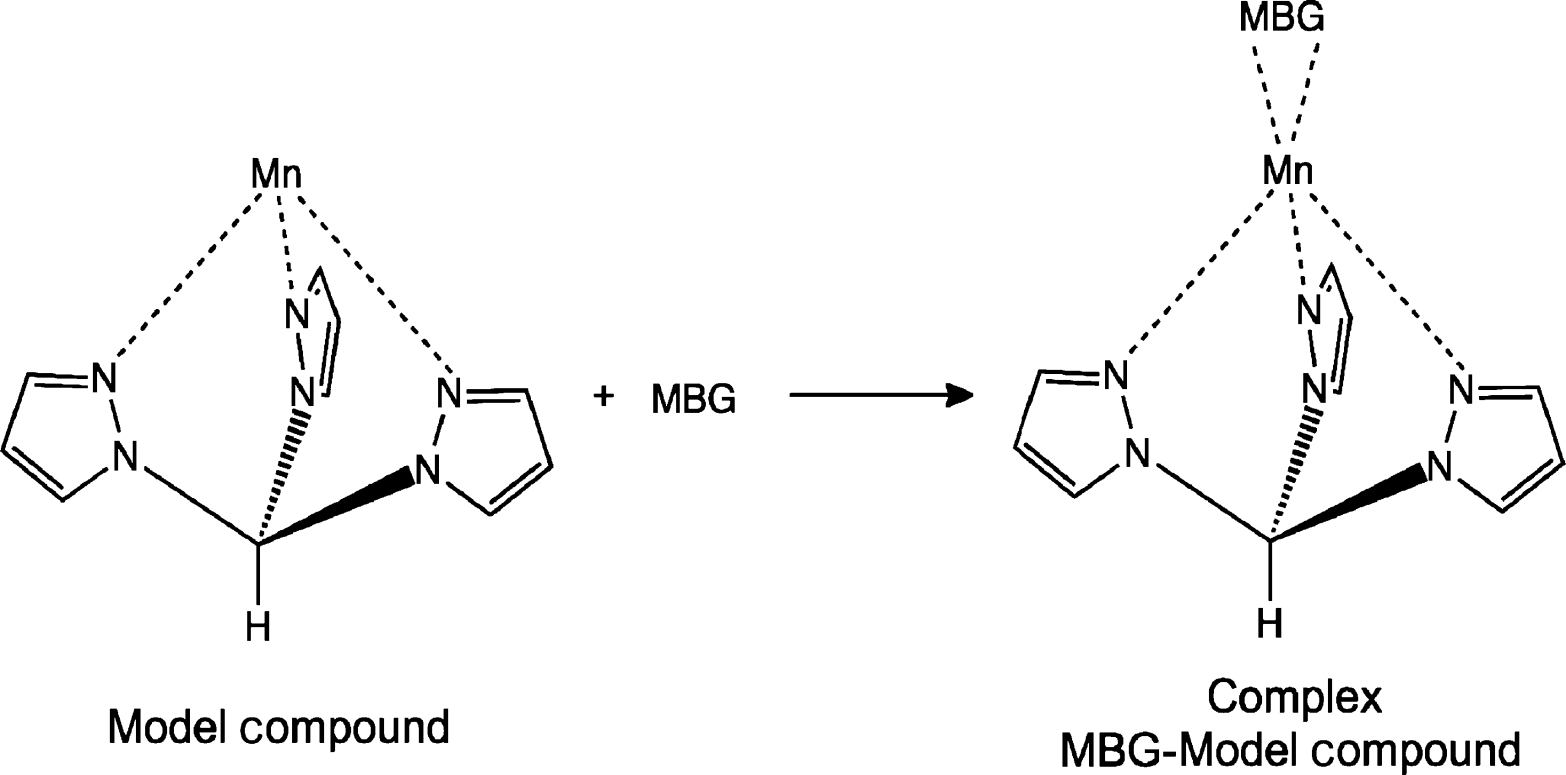
Model system used in the quantum mechanics study to calculate the binding energy of each metal-binding groups (MBGs 1–11, see Figure 1) to the model compound.

For this purpose, the affinity between the Mn^2+^ ion and the ligand was measured in the absence of the protein environment, using a Mn^2+^-containing scaffold molecule, based on a Mn^+2^ complex synthesized by Nabika *et al.* (21) (Figure 2), [Mn(II) tris(3.5-diisopropyl-1-pyrazolyl) methane]. This scaffold and the 12 compounds shown in Figure 1 were constructed using a drawing program available in Maestro. The ligand preparation wizard was used to add hydrogen atoms to each of the 12 compounds, with the hydroxyl groups assumed to be deprotonated at the protein active site at a pH of 7. Prior to calculations, each of the compounds was positioned at the same distance from the Mn^2+^ ion, as observed in the crystal structure of the *Mt*DXR enzyme when bound to fosmidomycin (PDB ID: 2JCZ).

For the geometry optimization, a BL3YP level of theory was applied, and for the energy calculations, LMP2/LACVP*+ level of theory was used, with the energies being counterpoise-corrected.

### Docking calculations

#### Generation of different active site conformations of *Mt*DXR

Each of the 12 compounds shown in Figure 1 was computationally docked into the active site of monomer A of the *Mt*DXR crystal structure (Table 2, PDB ID: 2JCZ), and three other conformations (labeled A-C in Table 2) selected from a prior study which examined the dynamics of the enzyme using an enhanced sampling MD technique (22). These three conformations were identified by clustering analysis as being the dominant conformations sampled throughout the simulations, together accounting for almost ∼70% of the total number of conformations sampled (see reference (22) for details). The active site of the crystal structure that is bound with the fosmidomycin inhibitor (PDB ID: 2JCZ, monomer A) is observed to be the most closed of the four conformations shown here, with an important catalytic loop overhanging and enclosing the binding site to the greatest extent. Structures A and B represent the monomer in conformations intermediate between the closed crystal structure and the most open form observed in Structure C (see Table 2), where the catalytic loop has moved away, exposing the active site.

#### QM-polarized docking calculations

In this study, the QM-polarized ligand-docking protocol of the Schrödinger suite (23) was utilized to dock the 12 MBGs into the active site of the four conformations of *Mt*DXR. This protocol improves docking accuracy over the non-QM docking, through the use of QM calculations (QSite package in Maestro) to determine the partial charges on the ligand atoms in the field of the receptor molecule, thus accounting for charge polarization. The purpose of this docking study was to determine the orientation of the ligands within the active site and predict any potential interactions with neighboring residues. The compound labeled ‘AHA’ (Figure 1) is the hydroxamate template ligand and is used as a reference compound for the docking of the 11 alternative metal-chelating groups studied here. The metal-chelating properties of the hydroxamate group are well established, and crystal structures observe the *Mt*DXR enzyme inhibitor, fosmidomycin, to form bidentate chelates between the inhibitor hydroxamate moiety and the active site metal ion (24–26).

Prior to the calculation, each of the protein and ligand structures was prepared, with hydrogens added according to the expected protonation at pH 7, using the protein preparation and ligand preparation wizards available within Maestro. A grid of an appropriate size, which fully incorporated the active site of the enzyme, was applied as the target area for the docking calculations.

The QM-polarized docking protocol initially docked each of the ligands using Glide before deriving the charges of the five top ligand poses in the field of the receptor, using QM calculations (QSite). In the final stage of the protocol, these poses are redocked into the active site of the receptor with the new charges, and the top 10 scoring poses were recorded.

## Results

One of the most frequently applied strategies in the development of metalloproteinase inhibitors consists in the design of molecules that contain a metal-chelating group (MBG) and backbone fragments (27). The backbones are drug-like structures that interact with active site binding pockets through non-covalent interactions, conferring selectivity toward a specific metalloprotein receptor. In recent years, Cohen and collaborators have expanded the library of MBGs for zinc-containing enzymes by introducing a new bioinorganic approach to design new matrix metalloproteinase inhibitors (20,28–35). The main idea behind the bioinorganic approach is to use small molecule complexes to model the active site of metalloenzymes. The complex [(Tp^Ph,Me^)Zn(OH)] (Tp^Ph,Me^ = hydrotris(3,5-phenylmethylpyrazolyl)borate) has been successfully applied to model the active site of zinc(II)-dependent MMP (20,31). The hypothesis is that as the three pyrazole rings mimic the catalytic histidines bound to the zinc in the MMP active site, the characterized complexes formed between the MBG and [(Tp^Ph,Me^)Zn(OH)] reflect the binding mode of the chelating group. This assumption has been supported by comparing the complex of [(Tp^Ph,Me^)Zn(AHA)], where AHA stands for acetohydroxamic acid, with the available crystal structures of the complexes formed between MMPs and hydroxamate-based inhibitors (31). In addition, by using this approach, the Cohen group was able to propose new classes of matrix metalloproteinase inhibitors exhibiting improved potency and novel selectivity relative to similar hydroxamic-based inhibitors against MMPs and anthax lethal factor (35–37).

Although several zinc-binding groups have been proposed in the literature, hydroxamic acid functional groups are still by far the most used MBG in MMP inhibitors (27). Similarly, fosmidomycin and its analogs also display the hydroxamate moiety in their structure. However, as aforementioned, hydroxamate-based MMP inhibitors are often associated with poor pharmacokinetics, poor oral bioavailability and toxicity (38–40). Monoanionic bidentate zinc-chelators such as pyrones, hydroxypyridinones, and their thione analogs (Figure 1) were the first heterocycles reported in the literature that showed significantly improved potency against MMPs when compared to acetohydroxamic acid (32,41). These compounds have been considered as very promising alternatives to hydroxamic acid owing to their low toxicity and relatively high potency against MMPs in cell culture (41).

Although some hydroxypyrone and hydroxypyridinone MBGs can modulate selective inhibition of MMPs when linked to biphenyl backbone fragments (42), it is usually assumed that the chelating group itself does not contribute significantly to selectivity. Because the main function of the MBG in the inhibitor molecules is to co-ordinate the catalytic metal, it has been proposed that the chelating group mainly contributes to the binding affinity of the inhibitor–metalloprotein complex. The lack of selectivity of acetohydroxamic acid for metal ions, such as Zn^2+^ and Mn^2+^, is also shown by the presence of a hydroxamic acid functional group in both DXR and MMP inhibitors.

To our knowledge, neither experimental nor theoretical work has investigated the use of pyrone-based molecules as chelating groups for enzymes besides zinc-dependent metalloproteins. In this work, we estimate the affinity of these newly identified metal-chelating groups to Mn^+2^ through the use of QM calculations to evaluate their binding energies according to the scheme showed in Figure 2. Inspired by the bioinorganic approach of Puerta *et al.* (34), we use the Mn^2+^ complex with tris(3,5-diisopropyl-1-pyrazolyl)methane ligand as our model compound to mimic the active site of *Mt*DXR (21). To reduce computational cost, all isopropyl groups were substituted by hydrogen atoms. The relative affinity to Mn^+2^ (ΔMn^affinity^) of each compound was defined according to eqn 1, where the energy difference between the bound and unbound states was calculated for each MBG, relative to AHA.


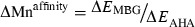
1

where Δ*E* is defined by,


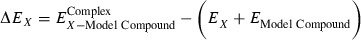
2

where *X* corresponds to a MBG structure in Figure 1.

Table 1 shows the binding affinity of each MBG, relative to AHA, obtained from the QM calculations made on the model system displayed in Figure 2. These results indicate that MBGs 1-11 have very similar affinity to Mn^2+^ when compared to AHA. Our QM calculations suggest that substitution of the hydroxamate moiety by any of the MBGs 1–11 can be well tolerated without compromising the inhibitor potency against *Mt*DXR. To further explore the use of MBGs 1–11 in the design of *Mt*DXR inhibitors, we carried out docking studies to evaluate their binding mode, metal chelation, and interactions with the *Mt*DXR active site resides.

**Table 1:** Relative Mn^affinity^ (Eqn 1) of proposed metal-binding groups

Metal-binding groups	ΔMn^affinity^ (fold)
1	1.0
2	0.9
3	1.1
4	0.9
5	0.9
6	0.9
7	0.9
8	0.9
9	1.1
10	0.9
11	0.9

Each of the ligands numbered **1–11** in Figure 1 and the reference compound, AHA, were docked into the four varied active site conformations (shown in Table 2) of monomer A of the *Mt*DXR enzyme, as described in the Methods section. The docking calculations showed that the majority of the compounds achieve a pose whereby the O, O (ligands **1–6**) or S, O (ligands **7–11**) donor atoms of the ligands form a bidentate chelate with the Mn ion, in a very similar orientation to what is observed with the hydroxamate moiety of the fosmidomycin inhibitor (and AHA reference compound) in the crystal structure. For a few of the ligands, a suitable docking pose was not achieved in all of the active site conformations, and this has been denoted by the absence of an entry in Table 2.

**Table 2:**
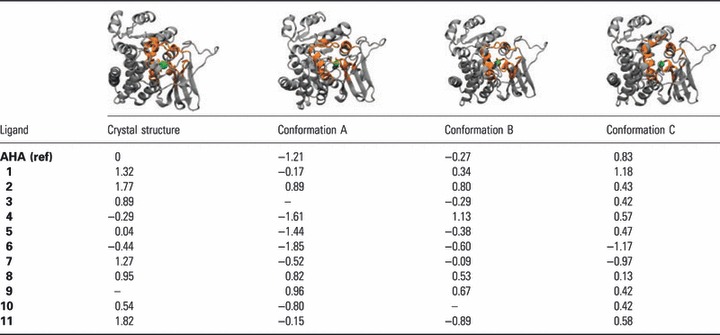
Relative binding affinity (ΔΔG^XP^ = docking score (Glide XP in kcal/mol) of acetohydroxamic acid (AHA) in the crystal structure – ligand docking score of AHA and compounds 1–11 into the crystal structure (monomer A of PDB ID: 2JCZ) and three predominant conformations sampled in a prior dynamics study (Conformations A–C). In a few cases, the ligands do not achieve acceptable docking poses where the ligand forms the bidentate chelate, and as a result, no docking score has been recorded (denoted as ‘–’)

**Figure 3:**
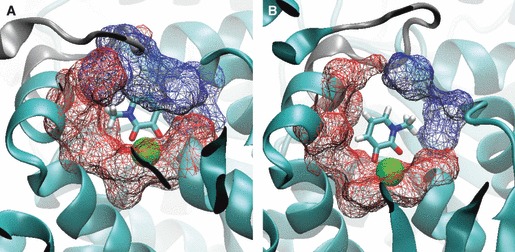
Docking pose of ligand 2 in the active site of (A) the closed crystal structure and (B) the more open Structure C. Residues shown as surface representation highlight the hydrophobic (blue) and polar (red) residues within 5 Å of ligand 6.

On docking each ligand into the four different *Mt*DXR conformations, variation is observed in the docking scores and poses owing to differences in active site volume of the various conformations and the orientations of residue side chains in the vicinity of the ligand. Generally, for ligands possessing bulky methyl groups, the space restrictions presented by some of the conformations represented in this study cause them to achieve lower docking scores than the non-methyl-containing ligands. This difference is most apparent when comparing the docking scores of methyl-containing ligands **2**, **3**, **8**, **9,** and **11**, when docked into the crystal structure, which has the most limited space within the active site, and Conformation C, which has the most available space of the four active site conformations. The steric constraints imposed by the more closed active sites recognize orientations of the ligand around the metal ion, which possess less favorable interactions with the residues of the active site. An example is shown in Figure 3, where ligand 2 flips orientation from its observed pose in the crystal structure active site conformation to that observed in Conformation C, where there is now available space to allow the methyl group to be positioned in a more hydrophobic neighborhood.

Of the 11 metal-chelating groups tested here, the non-methyl-containing ligands tend to observe improved binding affinity in the active site. Their best scores are observed in Conformation A, where the active site is less restricted compared with the crystal structure, but is still in a closed-type conformation, with the close packing of the active site residues providing a good environment for favorable interactions to exist. The consistently top ranked binding score of ligand 6 across the four *Mt*DXR conformations indicates that an amide group at a position adjacent to the metal-chelating carboxylate group on the ring may provide a favorable contribution to the binding affinity.

**Figure 4:**
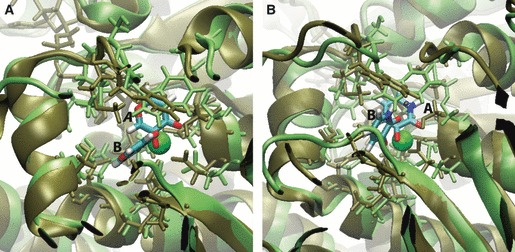
Docking orientations of (A) ligand 4 and (B) ligand 6 in Conformations A (yellow) and B (tan). Residue side chains of active site A are highlighted in ochra and those of active site B in green. The Mn^2+^ ion is shown as a green sphere, and the ligand poses are labeled A and B to indicate the pose observed active sites A and B, respectively.

All docked poses of the non-methyl-containing ligands exhibit the catalytically important Trp loop residue to be in close proximity and stacked over the ring of the ligand, providing the possibility for π–π interactions to occur. The docking poses of these ligands vary significantly, and of all the conformations, their least favorable docking scores are achieved with Conformation C. The reason for such differences in docking pose is illustrated in Figure 4, where the side chains of the hydrophobic Trp203 loop residue and Met205 are shown to impinge on the docking space of the active site in conformation B, sterically hindering the docking pose observed in the active sites of the crystal structure and Conformations A and C. This disrupts the favorable residue packing and opportunity for the π–π stacking between the ligand and Trp residue.

In the case of Conformation C, the docking scores of the non-methyl-containing ligands decrease slightly compared with Conformation A owing to the active site being significantly more open, with less opportunity for the ligand to interact with the more distant active site residues.

In summary, the docking calculations reveal the 11 zinc-binding moieties to interact with the Mn ion to form a bidentate chelate, with a similar or improved binding affinity compared with the hydroxamate reference compound. The results suggest the ligands without methyl substituents and possessing a polar group (e.g. amide) at a position adjacent to the metal-chelating moiety on the ring would provide improved interactions with the protein. In addition, the QM calculations showed that the binding energies between the Mn ion and compounds **1**–**11** are very similar to the one obtained for the AHA reference compound.

Therefore, from the computational studies performed here, the compounds are indicated to be promising candidates for experimental testing as viable alternatives to the hydroxamate group for a new class of inhibitors against the DXR enzyme in *M. tuberculosis.*

## Conclusions

The results of this study suggest a series of MBGs as promising candidates for the development of novel and potent inhibitors against the crucial *Mt*DXR enzyme in *M. tuberculosis*. These pyrone-based structures, originally developed by the Cohen group as metal-chelating groups for the Zn-dependent metalloproteinases, are indicated by computational QM and docking studies, to be transferable to the Mn-containing *Mt*DXR enzyme.

Quantum mechanics calculations demonstrated the binding affinity of each of the MBGs to Mn^2+^, as part of a scaffold structure, to be comparable to the reference AHA compound, which is known to chelate both Mn and Zn ions. QM-polarized docking calculations revealed the MBGs to form bidentate chelates with the Mn ion, as observed with fosmidomycin in the crystal structure, with similar or improved binding affinity to the reference AHA compound. In addition to the crystal structure, this docking was performed using three dominant protein conformations, as identified by MD simulations, removing the bias generated by the fosmidomycin-preformed active site conformation of the crystal structure, and provides increased knowledge of favorable/unfavorable structural aspects of these MBGs, which may be useful in the further development of these compounds into specific and potent inhibitors of *Mt*DXR.
